# Endoscopic Submucosal Dissection for Large Colorectal Tumor in a Japanese General Hospital

**DOI:** 10.1155/2013/218670

**Published:** 2013-08-29

**Authors:** Ken Ohata, Kouichi Nonaka, Yohei Minato, Yoshitsugu Misumi, Tomoaki Tashima, Meiko Shozushima, Takahiro Mitsui, Nobuyuki Matsuhashi

**Affiliations:** Division of Gastroenterology, NTT Medical Center Tokyo, 5-9-22 Higashigotanda, Shinagawa-ku, Tokyo 141-8625, Japan

## Abstract

*Background and Aims*. Endoscopic submucosal dissection (ESD) is not widely used in large colorectal lesions because of technical difficulty and possible complications. We aimed to examine the efficacy and safety of ESD for large colorectal neoplasms. *Patients and Methods*. During the past 5 years, 608 cases of colorectal neoplasm (≧20 mm) were treated by ESD. They were divided into Group A (20–49 mm, 511 cases) and Group B (≧50 mm, 97 cases). *Results*. The average age, lesion size, and procedure time were 67.4 years, 30.0 mm, and 60.0 min in Group A, and they were 67.1 years, 64.2 mm, and 119.6 min in Group B. En bloc resection rates were 99.2% and 99.0% (*P* = 0.80), and complication rates were 4.1% and 9.9% (*P* = 0.03). Complications in Group A consisted of perforation (2.7%), bleeding (1.2%), and ischemic colitis (0.2%). Those in Group B were perforation (8.2%) and bleeding (1.0%). Two cases in Group A and none in Group B required emergency surgery for perforation. *Conclusions*. There was no difference in efficacy between Groups A and B. Complications were more frequent in Group B, but all perforations in Group B were successfully managed conservatively. ESD can be effective and safe for large colorectal tumors.

## 1. Introduction

More than 20 years have passed since the introduction of endoscopic mucosal resection (EMR) to the treatment of digestive tract tumors, and the endoscopic treatment is now widely performed for early digestive tract cancers including stomach esophageal, and colon cancers [1–4]. More recently, endoscopic submucosal dissection (ESD) has been developed as a new technique [5], and an en bloc endoscopic resection of large lesions and lesions with ulcer scars has become possible [6].

ESD is a minimally invasive treatment and enables the en bloc resection for early colorectal neoplasm. However, it is not widely used in the large neoplastic lesions because of technical difficulty and complications. It has been reported that the tumor size of 50 mm or large is an independent risk factor for complications [7].

We aimed to examine the safety, efficacy and complications of ESD for large colorectal neoplasms (larger than 20 mm) in a nonacademic hospital in Japan, retrospectively.

## 2. Patients and Methods

We have treated 608 cases of colorectal neoplasm (size ≧20 mm) from July 2007 to December 2012.

All cases were carried out with 1 expert and/or 5 novice endoscopists who had performed under expert's supervision. We have treated 608 cases of colorectal neoplasm (size ≧20 mm) from July 2007 to December 2012. We divided the cases into two groups by size: Group A (lesion size: 20–49 mm) and Group B (lesion size ≧50 mm) (Table 1). Written informed consent was obtained from all patients. We evaluated tumor size, macroscopic type, histology, procedure time, en bloc and curative resection rates, and complications (Table 1).

### 2.1. Procedure of ESD

Details of the procedure have been described elsewhere [8–11].  In brief, normal saline was preinjected into the submucosal layer of the colon to avoid subsequent injections of sodium hyaluronate solution into an inappropriate layer. Sodium hyaluronate (0.5%) was then injected to make a good protrusion of the targeted mucosa. By mixing a small amount of dye, the sodium hyaluronate can be distinguished easily from the noninjected area even after the preinjection of normal saline. A small amount of epinephrine was also mixed with sodium hyaluronate to diminish bleeding during the procedures.

A mucosal incision around the tumor was then made with either a dual knife (KD-650L/KD-650Q; Olympus) or a flex knife (KD-630L; Olympus). Before incising the entire circumference of the lesion, dissection of the submucosa was started from the area in which the mucosal incision was completed, prior to the flattening of the lifted area as the procedure progressed.

The principal knife used for the submucosal dissection was the same one as that used for the mucosal incision.

**Figure 1:**
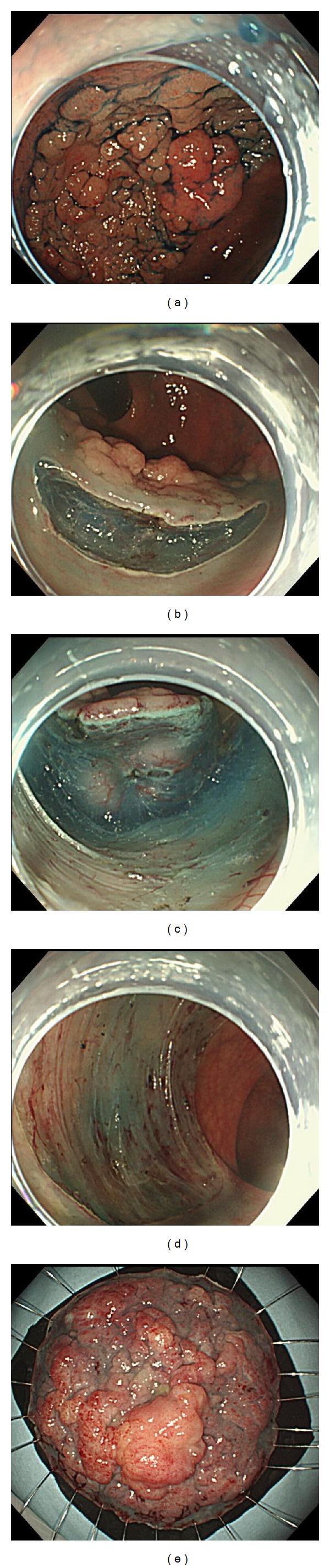
ESD for a 6.5 cm LST-G of the rectum: 6.5 cm LST-G is observed in the rectum.  Initial mucosal incision after submucosal injection at the oral side of the lesion. The body position was changed to allow the lesion to hang by gravity and, thus, to facilitate insertion of the endoscope into the submucosal layer. After the completion of ESD: about a 1/2 circumferential mucosal defect is observed: resected specimen.

The operation time was recorded for all the procedures. A typical example is shown in Figure 1.

CO_2_ insufflation was used instead of air insufflation. Since CO_2_ is absorbed more rapidly than air, it reduces the patient's discomfort due to an increase in gas in the intestine associated with a prolonged procedure, and if it should leak into the abdominal cavity due to perforation, it is absorbed relatively quickly.

ESD was performed under conscious sedation in the endoscopy room.

### 2.2. Histological Assessment

The specimens, fixed by formalin, were cut into 2 mm slices. They were examined microscopically for histological type, depth on invasion, lateral resection margin, and vertical resection margin. Histological assessments were based on the Japanese classification of cancer of colon and rectum and the Vienna classification [12–14]. Resections were considered tumor free when the lateral and vertical margins of a specimen were both negative for tumor cells independent of its histological features. A curative resection was achieved when both the lateral and the vertical margins of the specimen were free of cancer, and there was no SM invasion deeper than SM1, lymphatic invasion, vascular involvement, and poorly differentiated component. An adenoma with unknown lateral margin was also considered to be a curative resection provided that such adenoma met all of the other criteria. 

### 2.3. Statistical Analysis

All statistical analyses were performed by using JMP software version 8.0 (SAS Institute, Cary, NC, USA). Some variables in this study were described as mean (SD). The *P* value was 2 sided, and *P* < 0.05 was used to determine statistical validity. 

## 3. Results

For the 608 cases, 511 cases (84.0%) were assigned to group A, and 97 cases (16.0%) were assigned to Group B (Table 1). 

### 3.1. Clinicopathological Characteristics

The average age and the lesion size were 67.4 years and 30.0 mm in Group A, and 67.1 years, 64.2 mm respectively, in Group B.

Histologically, of the 511 tumors, there were 289 tubular adenomas (56.6%), 120 mucosal cancers (23.5%), 39 SM1 cancers (7.6%), 20 SM invasions 1000 *μ*m or more from the muscularis mucosae (SM2) or deeper (3.9%), and 43 serrated or nonneoplastic lesions (8.4%) in Group A. 

Macroscopic types included 260 nongranular-type LSTs (50.9%), 205 granular-type LSTs (40.1%), 40 protruded (7.8%), and 6 recurrent (1.2%) in Group A. 

On the other hand, of the 97 tumors, there were 43 tubular adenomas (44.3%), 28 mucosal cancers (28.9%), 8 SM1 cancers (8.2%), 10 SM invasions 1000 *μ*m or more from the muscularis mucosae (SM2) or deeper (10.3%), and 2 serrated or nonneoplastic lesions (2.1%) in group B.

Macroscopic types included 13 nongranular-type LSTs (13.4%), 80 granular-type LSTs (82.5%), 4 protruded (4.1%), and 0 recurrent (0%) in Group B. 

Tumor locations included 67 in the cecum (13.1%), 254 in the right colon (49.7%), 169 in the left colon (33.1%), and 88 in the rectum in Group A, and there were 17 in the cecum (17.5%), 49 in the right colon (50.5%), 18 in the left colon (18.6%), and 30 in the rectum (30.9%) in Group B. 

### 3.2. Clinical Outcomes of Colorectal ESD

The mean procedure time was 60 ± SD minutes in Group A, and it 119.6 ± SD minutes in Group B (*P* < 0.0001). The en bloc resection rate and the curative resection rate were 99.2% and 94.7% in Group A, and the 99.0% and 88.7% in Group B (*P* = 0.80 and  *P* = 0.12). There were statistically significant differences in the mean procedure time among 2 Groups (*P* < 0.0001). 

### 3.3. Complication Rate

Complications in Group A were 14 perforations (2.7%), 6 bleedings (1.2%), and 1 ischemic colitis (0.2%). In Group B were 8 perforations (8.2%) and 1 bleeding (1.0%) (*P* = 0.03).

Perforations during actual ESD procedures occurred in 13 patients (92.9%) in Group A and in 8 patients (100%) in Group B. Delayed perforations occurred in another 1 patient (7.1%) in Group A.

One delayed perforation and 1 immediate perforation required emergency surgery in Group A, but none in Group B.

## 4. Conclusions

 While esophageal or gastric neoplastic lesions undergoing endoscopic treatment are mostly early cancers, their colorectal counterparts are mostly benign (adenomatous). In addition, precise diagnostic techniques, including magnifying endoscopy, were established early on, facilitating the differentiation of adenomas from carcinomas and, preoperative estimation of the site and extent of the submucosal invasion with high-level accuracy [15, 16]. Based on the established preoperative diagnostic techniques, large lesions have been shown to be completely curable by divided endoscopic mucosal resection (EMR), which is currently performed worldwide. However, there are many lesions for which en bloc resection by ESD is desirable, such as large, depressed lesions untreatable by snare EMR, lesions strongly suspected of slight SM invasion before surgery, and lesions with fibrosis. Therefore, ESD, which has become a common technique for treating esophageal and gastric cancers, has recently come into use for the treatment of colorectal cancers. However, because ESD is associated with a high level of technical difficulty due to organ characteristics, and with frequent complications, colorectal ESD should be performed in high-volume endoscopy centers. 

 In our institution, a general hospital, the number of endoscopic procedures was so high that we had performed 608 colorectal ESDs up until December 2012.

 In 2010, Saito et al. [7] analyzed the results of more than 1,000 colorectal ESDs in 10 centers specialized for endoscopic treatment in Japan, and they reported that, in the 4 most experienced centers performing more than 100 colorectal ESDs, intraoperative perforation, delayed perforation, and postoperative bleeding occurred in 4.1%, 0.2%, and 1.1% of the patients, respectively. In the present study, intraoperative perforation, delayed perforation, and postoperative bleeding occurred in 3.5%, 0.2%, and 1.1% of the patients, respectively, which were compared favorably with the results for the above mentioned centers that are specialized in endoscopic treatment. In addition, the en bloc resection rate, the curative resection rate, and the procedure time in our hospital were 99.2%, 93.8%, and 73.0 min, respectively. These results were very favorably compared with those (89.0%, 89.7%, and 117 ± 91 min, resp.) in the 4 most experienced centers.

 ESD, if performed under the supervision of an expert even in a general hospital, has become a safe treatment modality. Because Saito et al. reported that a tumor size ≥50 mm was an independent risk factor for the development of complications, we evaluated the outcome of the treatment for Group A cancers (with a tumor size of 20–49 mm) and Group B (with a tumor size ≥50 mm) cancers in this study, and we found that the perforation rate was significantly higher and the procedure time was significantly longer for group B than for Group A cancers. However, all perforations were successfully managed conservatively, requiring no emergency surgery.

 Similar to the study of Saito et al., ESD for large colorectal neoplasms ≥5 cm was technically more difficult than that for their smaller counterparts, and it was associated with a high incidence of complications, all of which were successfully treated conservatively. Considering a procedure time of about 2 hours and the invasiveness of the surgery, it is necessary to further study the possibility of using ESD as a treatment option. In particular, extensive rectal lesions may require colostomy, giving a marked advantage to ESD. The present study led us to consider that, in a general hospital like ours, colorectal ESD can be performed relatively safely after training by, and under the supervision of, an experienced specialist.

 Although further studies involving more patients are needed, colorectal ESD seems to be a relatively safe and effective treatment for large (larger than 20 mm) superficial colorectal tumors.

## Figures and Tables

**Table 1:** Clinical characteristics of 608 colorectal ESDs divided into 2 separate groups.

	Group A (20–49 mm)	Group B (≧50)
Total ESDs	511	97
Age, y.o., mean ± SD	67.4 ± 10.3	67.1 ± 11.7
Tumor size, mm, mean ± SD	30.0 ± 7.50	64.2 ± 16.0
Tumor location		
Cecum	67	17
Right colon	254	49
Left colon	169	18
Rectum	88	30
Macroscopic type		
LST-G	205	80
LST-NG	260	13
Protruded	40	4
Recurrent	6	0
Histology		
Adenoma	289	43
Mucosal cancer	120	28
SM1 cancer	39	8
SM2 cancer	20	10
Serrated or nonneoplastic lesions	43	2
En bloc resection rate, %	99.2	99.0
Curative resection rate, %	94.7	88.7
Procedure time, min, mean ± SD	60 ± 35.3	119.6 ± 60.0
Complication, no. (%)		
Immediate perforation	13 (2.5%)	8 (8.2%)
Delayed perforation	1 (0.2%)	0 (0%)
Bleeding	6 (1.2%)	1 (1.0%)
Others	1 (0.2%)	

ESDs: endoscopic submucosal dissections, LST-G: granular -type laterally spreading tumor, SD: standard deviation, SM1: submucosal invasion less than 1000 *μ*m from the muscularis mucosae, and SM2: submucosal invasion 1000 *μ*m or more from the muscularis mucosae.
